# Identification of Potential Key lncRNAs and Genes Associated with Aging Based on Microarray Data of Adipocytes from Mice

**DOI:** 10.1155/2016/9181702

**Published:** 2016-12-21

**Authors:** Yi Yang, Zongyan Teng, Songyan Meng, Weigang Yu

**Affiliations:** Department of Geriatrics, The Second Affiliated Hospital of Harbin Medical University, Harbin, Heilongjiang 150086, China

## Abstract

*Objective*. This study aimed to screen potential crucial lncRNAs and genes involved in aging.* Methods*. The data of 9 peripheral white adipocytes, respectively, taken from male C57BL/6J mice (6 months, 14 months, and 18 months of age) in GSE25905 were used in this study. Differentially time series expressed lncRNA genes (DE-lncRNAs) and mRNA genes (DEGs) were identified. After cluster analysis of lncRNAs expression pattern, target genes of DE-lncRNAs were predicted from the DEGs, and functional analysis for target genes was conducted.* Results*. A total of 8301 time series-related DEGs and 43 time series-related DE-lncRNAs were identified. Among them, 41 DE-lncRNAs targeted 1880 DEGs. The DEGs positively regulated by DE-lncRNAs were mainly related to the development of blood vessel and the pathways of cholesterol biosynthesis and elastic fibre formation. Furthermore, the DEGs negatively regulated by DE-lncRNAs were correlated with protein metabolism.* Conclusion*. These DE-lncRNAs and DEGs are potentially involved in the process of aging.

## 1. Introduction

Aging is an elevated risk of common diseases, including obesity, hypertension, atherosclerosis, and diabetes [1–3]. Currently, about 800 million people are at least 60 years old, which accounts for about 11% of the world's population, and aging population is estimated to increase to more than 2 billion by 2050 [4]. Aging is closely related to damaged adipogenesis in various fat depots in humans [5, 6]. White adipose tissue (WAT) is considered as an important regulator for multiple physiological processes and highly linked to the development of multiple morbidities [7–9]. Therefore, it is significant and urgent to reveal the relationships of aging and adipose, which is very important for understanding the diseases in the elderly.

Previous studies have discovered a set of genes that are implicated in the aging process in an adipose depot-dependent manner. For example, age-related increase in* IL-6* (interleukin 6), which was related to stress responses and cellular senescence, was observed in a fat depot-dependent manner [5].* Sirt1* (sirtuin 1) and* SOD2* (superoxide dismutase 2), which were correlated with mitochondrial aging, were significantly decreased in aging epididymal adipocytes [10]. Furthermore, the expression of* MMP-3* (matrix metallopeptidase 3) was increased in mouse subcutaneous fat cells and human skin fibroblasts with aging [11, 12]. Additionally, decreased expression in* PPARγ* (peroxisome proliferator-activated receptor gamma) through declining fat mass has been observed in monkey subcutaneous whole fat tissue [13]. In addition to these genes mentioned above, roles of long noncoding RNAs (lncRNAs) in age-related diseases have attracted more attention recently [14, 15]. LncRNAs are defined as the largest transcript class in human genome longer than 200 bp that lack protein-coding potential [16, 17]. In aging murine aortas, mitochondrial lncRNA ASncmtRNA-2 is induced by replicative senescence [18]. Abnormal expression of the telomeric repeat-containing RNA lncRNA* TERRA* is responsible for premature senescence and aging through controlling telomere elongation [19]. In spite of much effort, the lncRNAs with known functions involved in aging remain rare.

Microarray technology has been widely used in molecular studies of human diseases [20, 21]. Based on an age-related gene expression profile GSE25905, Liu et al. have found high expression of genes involved in inflammatory response and low adipose-specific gene expression in bone marrow adipocytes, and age has a greater influence on gene expression in epididymal adipocytes than bone marrow adipocytes [22]. However, the effect of aging on expression of lncRNAs is still elusive.

In the current study, to investigate the expression variation and functional roles of lncRNAs in aging, the microarray data deposited by Liu et al. [22] were used to identify the differentially time series expressed lncRNA genes (DE-lncRNAs) and differentially time series expressed mRNA genes (DEGs) in the process of aging. Additionally, DEGs targeted by DE-lncRNAs and their functions were analyzed. The results may provide new information for the molecular investigation of aging and a deeper insight into aging.

## 2. Methods and Materials

### 2.1. Tissue Samples and Data Acquisition

The gene expression profile GSE25905 [22] was downloaded from the Gene Expression Omnibus (GEO, http://www.ncbi.nlm.nih.gov/geo/) database in National Center for Biotechnology Information (NCBI). The microarray data were produced on GPL6246 platform ([MoGene-1_0-st] Affymetrix Mouse Gene 1.0 ST Array, Affymetrix, CA, USA). This dataset contained 9 bone marrow adipocyte samples and 9 peripheral white adipocytes, respectively, taken from male C57BL/6J mice (6 months, 14 months, and 18 months of age), with three replicates at each age point. In this study, only gene microarray data of peripheral white adipocytes were used for further analysis.

### 2.2. Data Preprocessing

The gene expression profile of GSE25905 was preprocessed by the Robust Microarray Analysis (RMA) algorithm [23]. The Affy package (available at http://master.bioconductor.org/packages/release/bioc/html/affy.html) [24] of R. Probe IDs in CEL document was translated to corresponding gene symbols. If one gene symbol was matched by multiple probe IDs, the mean expression value was selected as the expression level of this gene.

### 2.3. Identification of DE-lncRNAs and DEGs

Based on annotation information of lncRNAs in GENCODE (http://www.gencodegenes.org/) [25] and the array platform GPL6246, expression data of lncRNAs were obtained. Afterwards, the BETR (Bayesian Estimation of Temporal Regulation) algorithm in the BETR package (http://betterpackages.com/) [26] was applied to identify DE-lncRNAs and DEGs at the three time points, and this algorithm calculated the probability of differential expression for each gene. The probability >0.9 was set as the cut-off criterion.

### 2.4. Cluster Analysis of DE-lncRNAs Expression Pattern

Hierarchical clustering is an analytical tool applied to discover the closest associations between gene profiles and specimens under evaluation [27, 28]. In our study, the BHC (Bayesian Hierarchical Clustering) package (http://master.bioconductor.org/packages/release/bioc/html/BHC.html) [29] of R was utilized to perform clustering of DE-lncRNAs and construct the cluster heat map of DE-lncRNAs and samples.

### 2.5. Prediction of DE-lncRNA Target Genes

Pearson correlation coefficient (PCC) [30] was used to calculate the expression similarity of DE-lncRNAs and DEGs at different time points. For each pair of DE-lncRNA and DEGs, significant correlation pairs with |PCC| > 0.95 and *p* value < 0.05 were used to construct the DE-lncRNA/DEG regulatory network which was then visualized by Cytoscape (http://js.cytoscape.org/) [31].

Furthermore, DE-lncRNA target genes that were known to be associated with aging were identified based on the information in AGEMAP (Atlas of Gene Expression in Mouse Aging Project), which is a gene expression database for aging in mice (http://cmgm.stanford.edu/~kimlab/aging_mouse) [32]. Subsequently, the regulatory network of DE-lncRNAs and the known aging-related targets was visualized by Cytoscape.

### 2.6. Functional Analysis

Gene Ontology (GO) functional and pathway enrichment analyses for genes positively and negatively regulated by DE-lncRNAs were carried out using TargetMine ( http://targetmine.mizuguchilab.org/) [33]. The *p* value of each GO and pathway term was adjusted by the Holm-Bonferroni method [34], and adjusted *p* value < 0.05 was considered statistically significant. Additionally, the pathway network was constructed using Cytoscape.

## 3. Results

### 3.1. Identified DE-lncRNAs and DEGs

Based on the annotation information in GENCODE and GPL6246 platform, a total of 203 probes were annotated as lncRNA genes, and 20564 probes were annotated as mRNA genes. With the cut-off of probability >0.9, 8301 time series DEGs and 43 DE-lncRNAs were identified in peripheral white adipocyte samples.

### 3.2. Clusters of DE-lncRNAs Expression Pattern

**Figure 1:**
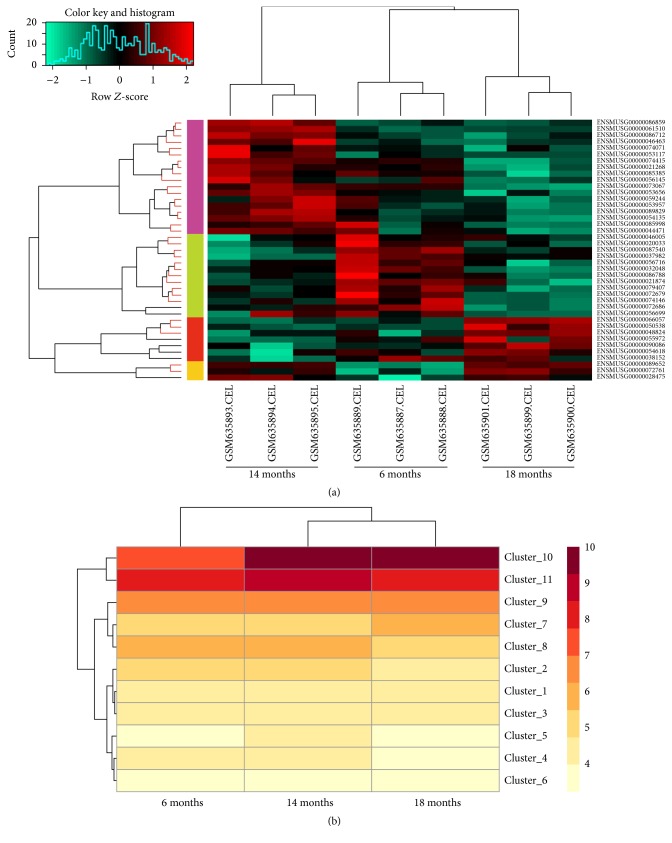
Heat maps of differentially expressed lncRNA genes. (a) Heat map of differentially expressed lncRNA genes in peripheral white adipocytes samples from male C57BL/6J mice being 6 months, 14 months, and 18 months of age. Each row represents a single gene; each column represents a sample. The gradual color change from red to green represents the changing process of expression level from upregulation to downregulation. (b) Heat map of clusters of differentially expressed lncRNA genes at 6, 14, and 18 months. Each row represents a cluster; each column represents a time point. LncRNA, long noncoding RNA.

To further explore the changes of the DE-lncRNAs expression levels at the three time points in peripheral white adipocytes, the cluster analysis was conducted. The samples at different time points were distinguished by DE-lncRNAs. The expression values of nearly half of DE-lncRNAs showed an uptrend in 6–14 months and a downtrend in 14–18 months (e.g., ENSMUSG00000086859 and ENSMUSG00000061510); a set of DE-lncRNAs were expressed in a decline trend (e.g., ENSMUSG00000087540 and ENSMUSG00000032048); and a small fraction of DE-lncRNAs were expressed in a rising trend (e.g., ENSMUSG00000066057) (Figure 1(a)).

According to the results of clustering analysis, 41 DE-lncRNAs were divided into 11 clusters (Table 1). It was clearly observed that, with the increase of age in mice, DE-lncRNAs in clusters 7 and 10 were expressed in a rising trend, whereas DE-lncRNAs in clusters 2, 4, and 8 were expressed in a decline trend. Clusters 5 and 11 showed an uptrend in 6–14 months and a downtrend in 14–18 months (Figure 1(b)).

### 3.3. DEGs Targeted by DE-lncRNAs

**Figure 2:**
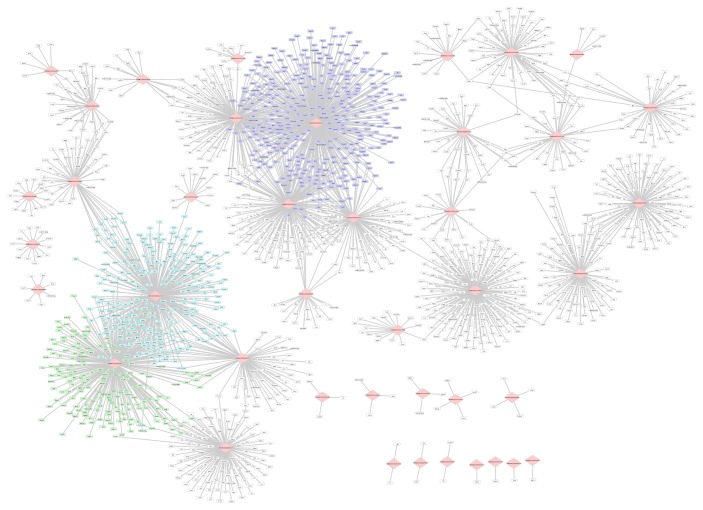
The regulatory network of 41 differentially expressed lncRNA genes and their target mRNA genes. The diamonds represent lncRNA gene IDs, and rectangles represent mRNA genes. The purple nodes represent the target genes of ENSMUSG00000066057; the blue nodes represent the target genes of ENSMUSG00000061510; the green nodes represent the target genes of ENSMUSG00000086859. LncRNA, long noncoding RNA.

**Figure 3:**
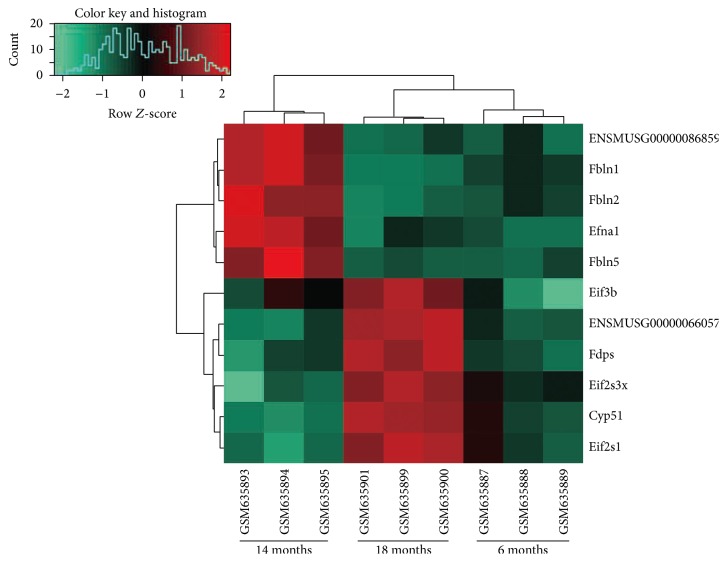
Heat map showing the expression changes over time of ENSMUSG00000086859 and ENSMUSG00000066057, as well as some targets of them. Each row represents a single gene or lncRNA; each column represents a sample. The gradual color change from red to green represents the changing process of expression level from upregulation to downregulation. LncRNA, long noncoding RNA.

LncRNAs have critical roles in the transcriptional regulation via modulating the gene expressions. To further investigate the regulatory functions of DE-lncRNAs, the DEGs regulated by DE-lncRNAs were analyzed by the PCC algorithm. Based on the cut-off criteria, 2313 regulatory relationships between DE-lncRNAs and DEGs were obtained (see Supplementary Material available online at http://dx.doi.org/10.1155/2016/9181702). The constructed regulatory network consisted of 41 DE-lncRNAs and 1880 DEGs. The DE-lncRNAs ENSMUSG00000066057, ENSMUSG00000086859, and ENSMUSG00000061510 modulated more DEGs than others. ENSMUSG00000086859 targeted genes like* Efna1*,* Fbln1*, and* Fbln2*. ENSMUSG00000066057 regulated the DEGs, such as* CYP51*,* FDPS*, and* Eif2s1* (Figure 2). The expression changes over time of ENSMUSG00000086859 and ENSMUSG00000066057, as well as some targets of them, were shown in Figure 3.

**Figure 4:**
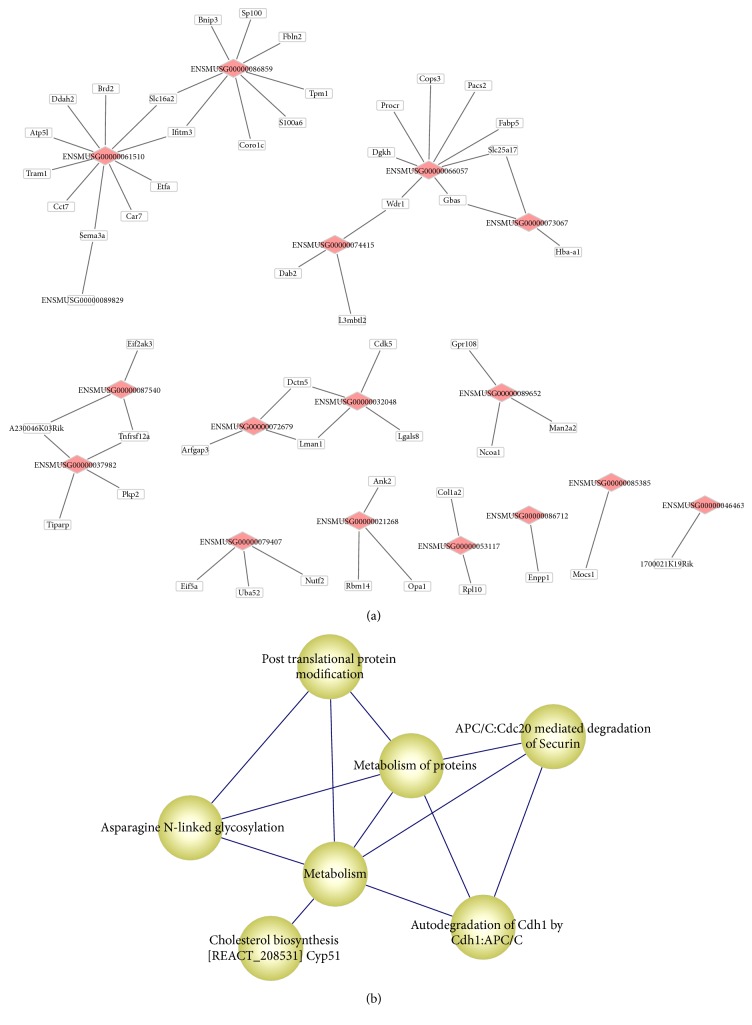
Networks of genes and pathways. (a) The regulatory network of differentially expressed lncRNA genes and age-related target genes. The diamonds represent lncRNA gene IDs, and rectangles represent mRNA genes. (b) The network of pathways that are enriched by target genes. Nodes represent pathways, and lines indicate that there are common genes related to the two pathways. The larger the thickness of line is, the more the common genes are. LncRNA, long noncoding RNA.

Based on the AGEMAP database, a total of 51 DE-lncRNA target genes had been discovered to be correlated with aging in mice. There were 16 DE-lncRNAs that regulated these genes, and 61 regulatory relationships were included in the network. Both ENSMUSG00000086859 and ENSMUSG00000061510 targeted* Slc16a2* and* Ifitm3*; ENSMUSG00000066057 regulated aging-related DEGs like* Wdr1* (Figure 4(a)).

### 3.4. Enrichment Analysis of DE-lncRNA Targets

To further reveal the potential functions mediated by DE-lncRNAs, the GO and pathway enrichment analyses of DE-lncRNA targets were performed, respectively. The DEGs positively regulated by DE-lncRNAs (e.g.,* Efna1* and* Efnb2*) were mainly enriched in a set of biology processes about the development of blood vessel, such as vasculature development and blood vessel morphogenesis (Table 2). A series of positively regulated target genes were significantly enriched in the pathways of cholesterol biosynthesis (e.g.,* Cyp51* and* Fdps*) and elastic fibre formation (e.g.,* Fbln1*,* Fbln2*, and* Fbln5*) (Table 2).

Furthermore, the negatively regulated target genes of DE-lncRNAs were mainly enriched in a set of biology processes, such as metabolic process (e.g.,* Abi3* and* Acaca*) and mitochondrion organization (e.g.,* Acaa2* and* Bnip3*), as well as pathways like metabolism of proteins (e.g.,* Eif2s1*,* Eif2s3x*, and* Eif3b*) (Table 3).

Additionally, the pathway of metabolism of proteins was predicted to interact with five other pathways, such as posttranslational protein modification and asparagine N-linked glycosylation (Figure 4(b)).

## 4. Discussion

The increased occurrence of age-related diseases, such as cancers, chronic inflammatory, and neurodegenerative diseases, becomes a burden on health care provision in the developed and developing countries [35]. In this study, gene expression profile GSE25905 was downloaded and analyzed using bioinformatics methods to explore the potential mechanisms of aging. A total of 8301 time series DEGs and 43 time series DE-lncRNAs were identified in peripheral white adipocyte samples. In the DE-lncRNAs/DEGs regulatory network, the DE-lncRNAs ENSMUSG00000066057, ENSMUSG00000086859, and ENSMUSG00000061510 regulated multiple DEGs. The DEGs positively regulated by DE-lncRNAs were mainly enriched in the functions about the development of blood vessel (e.g.,* Efna1* and* Efnb2*), as well as the pathways of cholesterol biosynthesis (e.g.,* Cyp51* and* Fdps*) and elastic fibre formation (e.g.,* Fbln1*,* Fbln2*, and* Fbln5*).

The function of blood vessel development was significantly enriched by a set of DE-lncRNA genes, such as* Efna1* and* Efnb2*. During aging, angiogenesis is delayed, and capillary density as well as newly deposited collagen is decreased [36]. Cardiovascular structure and function are altered during aging, with elongated and stiffer aorta, as well as changed arterial baroreflex [37]. Both* Efna1* and* Efnb2* encode members of the ephrin family, which mediates developmental events [38]. It has been confirmed that the balance of alternatively expressed isoforms in* Efna1* is disrupted in peripheral blood leukocytes of human population with advancing age [39]. Furthermore, the expression of* Efnb2* is significantly decreased during the aging of the rat retina [40]. In this study,* Efna1* was predicted to be regulated by the DE-lncRNA ENSMUSG00000086859 (gene name: 2810008D09Rik). There is no study that reports the role of ENSMUSG00000086859 in aging so far. Collectively, we speculate that ENSMUSG00000086859 may play key roles in aging through genes related to blood vessel development (e.g.,* Efna1*).

In this study, several other DEGs positively regulated by DE-lncRNAs (e.g.,* Fbln1*,* Fbln2,* and* Fbln5*) were significantly enriched in the pathway of elastic fibre formation. During cutaneous aging, elastic fibres exhibited disintegration and appeared to be loose [41]. All of* Fbln1*,* Fbln2*, and* Fbln5* encode a secreted glycoprotein that is incorporated into a fibrillar extracellular matrix [42]. During aging, the balance between proteases and their inhibitors involved in extracellular matrix formation is destroyed [43]. In this study,* Fbln1* and* Fbln2* were discovered to be an age-regulated gene and regulated by the DE-lncRNA ENSMUSG00000086859 (gene name: 2810008D09Rik). The association of* Fbln2* with human aging has also been discovered by previous studies [44, 45]. Moreover,* Fbln5* was predicted to be targeted by ENSMUSG00000061510. Therefore, ENSMUSG00000086859 may also exert functions in aging via regulating the genes involved in elastic fibre formation (e.g.,* Fbln1* and* Fbln2*). ENSMUSG00000061510 may function in aging via regulating the expression of genes like* Fbln5*.

A previous study has demonstrated that aging is associated with altered cholesterol metabolism in T cells, causing increased cholesterol levels in lipid rafts [46]. Furthermore, cholesterol transport and lipid catabolism have been identified to be upregulated in normally aging rats [47, 48]. In the present study,* CYP51* and* FDPS*, the positively regulated target genes of the DE-lncRNA ENSMUSG00000066057 (gene name: Gm1976), were significantly enriched in cholesterol biosynthesis.* CYP51*, the most evolutionarily conserved member of cytochrome P450 gene superfamily, participates in the late portion of cholesterol biosynthesis [49]. In aging peripheral nervous system and liver,* CYP51* is also detected to be involved in the deregulation of cholesterol biosynthesis [50, 51].* FDPS* encodes farnesyl diphosphate synthase, which is a key intermediate in cholesterol and sterol biosynthesis [52]. Previous studies have reported that* FDPS* is associated with bone mineral density of aging bone [53, 54]. During aging, cholesterol synthesis is reduced in human hippocampus [55]. For example, the concentration of three cholesterol precursors (lathosterol, lanosterol, and desmosterol) is significantly decreased in the hippocampus [56]. Currently, there is no experimental evidence that ENSMUSG00000066057 is involved in aging. Therefore, this DE-lncRNA may play a role in aging via the genes related to cholesterol synthesis (e.g.,* Cyp51* and* Fdps*).

Furthermore, the DEGs that were negatively regulated by ENSMUSG00000066057 (e.g.,* Eif2s1*) were mainly enriched in the pathways about protein metabolism along with* Eif2s3x* and* Eif3b*, and several pathways about protein metabolism interacted with each other. In aging humans, the balance between muscle protein synthesis and degradation is disrupted, which leads to the loss of skeletal muscle mass [57]. All of* Eif2s1*,* Eif2s3x*, and* Eif3b* encode subunits of eukaryotic translation initiation factors (EIFs), which regulate protein synthesis [58]. Decreased eIF2*α* phosphorylation has been detected in aged tissues and it is responsible for a higher level of protein phosphatase 1 and other proapoptotic proteins [59, 60]. There is no evidence to prove the roles of* Eif2s1*,* Eif2s3x*, and* Eif3b* in aging so far. We speculate that the ENSMUSG00000066057 may also play critical roles in aging via regulating protein metabolism through* Eif2s1*. The DEGs* Eif2s1*,* Eif2s3x*, and* Eif3b* may also be involved in aging via protein metabolism.

Despite the aforementioned results, there were several limitations in this study. The predicted results should be confirmed by laboratory data. Furthermore, the included samples for analysis should be more. In our further studies, more samples of aging will be included to validate the expression levels and functions of the potential key lncRNAs and genes.

In conclusion, based on the gene expression data of peripheral white adipocytes taken from mice at different ages, a total of 8301 time series DEGs and 43 time series DE-lncRNAs were identified. Among them, 41 DE-lncRNAs targeted 1880 DEGs. The DE-lncRNAs ENSMUSG00000066057, ENSMUSG00000086859, and ENSMUSG00000061510 regulated multiple DEGs. Furthermore, the DEGs positively regulated by DE-lncRNAs (e.g., ENSMUSG00000066057 and ENSMUSG00000086859) were mainly related to the functions about the development of blood vessel (e.g.,* Efna1* and* Efnb2*), as well as the pathways of cholesterol biosynthesis (e.g.,* Cyp51* and* Fdps*) and elastic fibre formation (e.g.,* Fbln1*,* Fbln2*, and* Fbln5*). Additionally, the DEGs (e.g.,* Eif2s1*,* Eif2s3x*, and* Eif3b*) that were negatively regulated by DE-lncRNAs were correlated with the pathways about protein metabolism. These DE-lncRNAs and DEGs may be involved in aging, which provides novel information for the study of aging.

## Supplementary Material

The regulatory relationships between differentially time series expressed lncRNA genes and mRNA genes.

## Figures and Tables

**Table 1:** Differentially time-series expressed long noncoding RNA genes in each cluster.

Cluster number	Count	Differentially time-series expressed long noncoding RNA gene ID
1	3	ENSMUSG00000079407, ENSMUSG00000072686, ENSMUSG00000032048
2	2	ENSMUSG00000072679, ENSMUSG00000073067
3	8	ENSMUSG00000056145, ENSMUSG00000090086, ENSMUSG00000074071, ENSMUSG00000046463, ENSMUSG00000055972, ENSMUSG00000053957, ENSMUSG00000048824, ENSMUSG00000053117
4	6	ENSMUSG00000046005, ENSMUSG00000021874, ENSMUSG00000056699, ENSMUSG00000059244, ENSMUSG00000056716, ENSMUSG00000086788
5	1	ENSMUSG00000061510
6	3	ENSMUSG00000087540, ENSMUSG00000074146, ENSMUSG00000020033
7	4	ENSMUSG00000050538, ENSMUSG00000054618, ENSMUSG00000038152, ENSMUSG00000089829
8	6	ENSMUSG00000054135, ENSMUSG00000053656, ENSMUSG00000086859, ENSMUSG00000086712, ENSMUSG00000074415, ENSMUSG00000021268
9	6	ENSMUSG00000085385, ENSMUSG00000066057, ENSMUSG00000037982, ENSMUSG00000028475, ENSMUSG00000085998, ENSMUSG00000072761
10	1	ENSMUSG00000089652
11	1	ENSMUSG00000044471

**Table 2:** Results of Gene Ontology functional and pathways enrichment analyses for genes positively regulated by differentially time-series expressed lncRNAs.

Category	Term	Adjust *p* value	Gene count	Genes
GO-BP	Vasculature development [GO:0001944]	1.33*E* − 08	65	*Adamts1*,* Cdh5*,* Ctsh*,* Cxcr3*,* Dhcr7, Edn1*, *Efna1*, *Efnb2*, *Notch3*, *Pdgfrb*, and so on
GO-BP	Blood vessel development [GO:0001568]	1.34*E* − 07	60	*Adamts1*, *Cdh5*, *Ctsh*, *Cxcr3*, *Dhcr7*, *Edn1*, *Efna1*, *Efnb2*, *Notch3*, *Pdgfrb,* and so on
GO-BP	Regulation of locomotion [GO:0040012]	2.45*E* − 05	51	*Dab2*, *Ifitm3*, *Il16*, *Il33*, *Irs2*, *Megf8*, *Myo1f*, *Pdgfrb*, *Pecam1*, *Pkn1,* and so on
GO-BP	Blood vessel morphogenesis [GO:0048514]	2.55*E* − 05	49	*Adamts1*, *Aqp1*, *C3*, *Ccr2*, *Edn1*, *Efna1*, *Efnb2*, *Egfl7*, *Elk3*, *Ephb4,* and so on
GO-BP	Regulation of cellular component movement [GO:0051270]	3.92*E* − 05	50	*Ccl21a*, *Dab2*, *Ddr2*, *Dpep1*, *Efna1*, *Nup155*, *Pde4d*, *Pdgfra*, *Pdgfrb*, *Pecam1,* and so on
GO-CC	Cell surface [GO:0009986]	2.46*E* − 05	53	*Ackr3*, *Alcam*, *Cd200r1*, *Cd3e*, *Dpp4*, *Enpp1*, *Flt3l*, *Heg1*, *Ifitm3*, *Il2rb,* and so on
GO-CC	Plasma membrane [GO:0005886]	9.39*E* − 05	158	*Ano1*, *Antxr1*, *Aqp1*, *Capn3*, *Ccr2*, *Itga5*, *Itm2c*, *Kcnab1*, *Kcnn3*, *Kcnt2,* and so on
GO-CC	Cell periphery [GO:0071944]	2.59*E* − 04	163	*Antxr1*, *Aqp1*, *Bcas3*, *Capn3*, *Eps15l1*, *Ezr*, *Krt19*, *Lime1*, *Ntn4*, *P2rx4,* and so on
GO-CC	Side of membrane [GO:0098552]	0.015056	33	*Alcam*, *Ano1*, *Cd74*, *Ikbkb*, *Il2rb*, *Itga5*, *Kdr*, *Ly6a*, *Ly6c1*, *Pkp4,* and so on
GO-CC	Plasma membrane part [GO:0044459]	0.021127	101	*Klri1*, *Lime1*, *Ly6a*, *Npc1*, *P2rx4*, *Sema6a*, *Sept2*, *Tspan15*, *Upk1b*, *Zdhhc2,* and so on
REACT_208531	Cholesterol biosynthesis	4.00*E* − 03	9	*Cyp51*, *Dhcr7*, *Fdps*, *Hsd17b7*, *Idi1*, *Lss*, *Mvk*, *Sc5d*, *Sqle*
REACT_198996	Elastic fibre formation	5.10*E* − 03	11	*Efemp1*, *Efemp2*, *Fbln1*, *Fbln2*, *Fbln5*, *Furin*, *Itga5*, *Loxl1*, *Ltbp1*, *Mfap3,* and so on

The GO-BP terms in the table are the top 5 ones with a higher adjusted *p* value. DE-lncRNA, differentially time-series expressed long noncoding RNA gene; GO, Gene Ontology; BP, biological process; CC, cellular component. “REACT” terms are the pathway terms.

**Table 3:** Results of Gene Ontology functional and pathways enrichment analyses for genes negatively regulated by differentially time-series expressed lncRNAs.

Category	Term	Adjust *p* value	Gene count	Genes
GO-BP	Metabolic process [GO:0008152]	8.05*E* − 10	380	*Abi3*, *Acaca*, *Acadvl*, *Brd2*, *Capn15*, *Cxcl9*, *D2hgdh*, *Dagla*, *Gemin5*, *Gfm1,* and so on
GO-BP	Organic substance metabolic process [GO:0071704]	5.74*E* − 09	360	*Acox1*, *Acsm3*, *Capn15*, *Casp2*, *Ddx20*, *Decr1*, *Dennd3*, * Frzb*, *Fxr1*, *Gadd45a,* and so on
GO-BP	Primary metabolic process [GO:0044238]	3.62*E* − 08	343	*Acadvl*, *Acot7*,* Babam1*, *Bach1*, *Eif3c*, *Ell*, *Gbp2*, *Gclm Mtdh*, *Nabp1,* and so on
GO-BP	Cellular metabolic process [GO:0044237]	5.03*E* − 08	344	*Acadvl*, *Acot7*, *Babam1*, *Bach1*, *Eif3c*, *Ell*,* Gbp2*, *Gclm Mtdh*, *Nabp1,* and so on
GO-BP	Mitochondrion organization [GO:0007005]	2.30*E* − 04	32	*Acaa2*, *Bnip3*, *Cln8*, *Dap3*, *March5*, *Mrpl44*, *Mtch2*, *Mtfr2*, *Ptcd2*, *Slc22a5,* and so on
GO-CC	Intracellular [GO:0005622]	2.01*E* − 16	478	*Abi3*, *Acaa2*, *Acyp2*, *Adap2*, *Capn15*, *Ggct*, *Ggh*, *Itgb1bp1*, *Jak2*, *Katna1,* and so on
GO-CC	Intracellular part [GO:0044424]	3.17*E* − 16	475	*Abi3*, *Acaa2*, *Acyp2*, *Adap2*,* Capn15*, *Ggct*, *Ggh*, *Itgb1bp1*, *Jak2*, *Katna1,* and so on
GO-CC	Cell [GO:0005623]	4.05*E* − 15	526	*Atp5l*, *B4galt1*,* Dusp8*, *Dynll2*,* Exoc4*, *Faf1*,* Hint2*, *Hmox1*, * Rtn2*, *S100a11,* and so on
GO-CC	Cell part [GO:0044464]	4.05*E* − 15	526	*Atp5l*, *B4galt1*, *Dusp8*, *Dynll2*, *Exoc4*, *Faf1*, *Hint2*, *Hmox1*, * Rtn2*, *S100a11,* and so on
GO-CC	Mitochondrion [GO:0005739]	7.42*E* − 12	134	*Atp5l*, *Bcs1l*, *Hspd1*, *Iars2*,* Ptrf*, *Rab11a*, *Sugct*, *Tango2*, * Trmt2b*, *Uqcc2,* and so on
GO-MF	Catalytic activity [GO:0003824]	1.50*E* − 03	180	*Abhd6*, *Acaca*, *Dusp8*, *Ebp*,* Htra1*, *Huwe1*, *Itpkc*,* Lypla1*, *Man2a1*, *Nek6,* and so on
REACT_188937	Metabolism	2.32*E* − 04	125	*Agpat4*, *Akr1c13*, *Cth*, *D2hgdh*, *Gstm7*, *Helz2*,* Ogn*, *Pank2*,* Psma1*,* Suclg2,* and so on
REACT_247926	Metabolism of proteins	0.004417	58	*Eif2s1*, *Eif2s3x*, *Eif3b*, *Hspd1*, *Igf1*, *Man2a1*, *Nfyc*, *Pam16*, *Rft1*, *Slc30a6,* and so on
REACT_237472	Asparagine N-linked glycosylation	0.004713	19	*Alg11*, *Gfpt2*, *Gnpnat1*, *Lman1*, *Mgat2*, *Rft1*, *Slc35a1*, *St3gal1*, *St6galnac5*, *Uap1,* and so on
REACT_236283	Posttranslational protein modification	0.010703	27	*Alg11*, *Eif5a*, *Galnt2*, *Gfpt1*, *Gfpt2*, *Man2a1*, *Senp5*, *Slc35a1*, *St3gal1*, *St6galnac5,* and so on
REACT_225686	Autodegradation of Cdh1 by Cdh1:APC/C	0.012911	13	*Cdc16*, *Cdc23*, *Cdc27*, *Psma1*, *Psma2*, *Psma4*, *Psmb4*, *Psmb8*, *Psmb9*, *Psmc6,* and so on
REACT_219897	APC/C:Cdc20 mediated degradation of Securin	0.027596	13	*Cdc16*, *Cdc23*, *Cdc27*, *Psma1*, *Psma2*, *Psma4*, *Psmb4*, *Psmb8*, *Psmb9*, *Psmc6,* and so on

The GO-BP and GO-CC terms in the table are the top 5 ones with a higher adjusted *p* value. DE-lncRNA, differentially time-series expressed long noncoding RNA gene; GO, Gene Ontology; BP, biological process; CC, cellular component. “REACT” terms are the pathway terms.
